# Elevated serum levels of human epididymis protein 4 in adult patients with proliferative lupus nephritis

**DOI:** 10.3389/fimmu.2023.1179986

**Published:** 2023-05-23

**Authors:** Liubing Li, Huiya Xu, Yuting Le, Runzhao Li, Qiong Shi, Hongji Zhu, Hongxu Xu, Laisheng Li, Min Liu, Fen Wang, Hui Zhang

**Affiliations:** ^1^ Department of Laboratory Medicine, The First Affiliated Hospital, Sun Yat-sen University, Guangzhou, China; ^2^ Department of Pathology, The First Affiliated Hospital, Sun Yat-sen University, Guangzhou, China; ^3^ Department of Rheumatology, The First Affiliated Hospital, Sun Yat-sen University, Guangzhou, China; ^4^ Institute of Precision Medicine, The First Affiliated Hospital, Sun Yat-sen University, Guangzhou, China

**Keywords:** serum HE4, diagnostic efficacy, proliferative lupus nephritis, pathological classes, active/chronic lesions

## Abstract

**Background:**

This study aimed to access whether serum human epididymis protein 4 (HE4) level could identify lupus nephritis (LN) pathological classes in adults and children.

**Methods:**

The serum HE4 levels of 190 healthy subjects and 182 patients with systemic lupus erythematosus (SLE) (61 adult-onset LN [aLN], 39 childhood-onset LN [cLN], and 82 SLE without LN) were determined using Architect HE4 kits and an Abbott ARCHITECT i2000SR Immunoassay Analyzer.

**Results:**

Serum HE4 level was significantly higher in the aLN patients (median, 85.5 pmol/L) than in the patients with cLN (44 pmol/L, *P* < 0.001) or SLE without LN (37 pmol/L, *P* < 0.001), or the healthy controls (30 pmol/L, *P* < 0.001). Multivariate analysis showed that serum HE4 level was independently associated with aLN. Stratified by LN class, serum HE4 level was significantly higher in the patients with proliferative LN (PLN) than in those with non-PLN, and this difference was found only in aLN (median, 98.3 *versus* 49.3 pmol/L, *P* = 0.021) but not in cLN. Stratified by activity (A) and chronicity (C) indices, the aLN patients with class IV (A/C) possessed significantly higher serum HE4 levels than those with class IV (A) (median, 195.5 *versus* 60.8 pmol/L, *P* = 0.006), and this difference was not seen in the class III aLN or cLN patients.

**Conclusion:**

Serum HE4 level is elevated in patients with class IV (A/C) aLN. The role of HE4 in the pathogenesis of chronic lesions of class IV aLN needs further investigation.

## Introduction

Systemic lupus erythematosus (SLE) is a chronic inflammatory autoimmune disease that can occur during childhood or adulthood and is characterized by multisystem and multiorgan involvement (1). Lupus nephritis (LN) is one of the most common and severe manifestations, notably in African, Asian, and Hispanic populations (2, 3), affecting 40–60% of patients with SLE (4, 5), and up to 30% of patients with LN progress to end-stage renal disease (ESRD) (6–10). LN patients show 6–26-fold mortality compared with the general population, and this disease has been a major cause of death in the patients (6, 11, 12).

According to the classification system of the International Society of Nephrology/Renal Pathology Society (ISN/RPS), LN is classified into six classes (13), of which proliferative LN (PLN; class III, class IV, class III+V, and class IV+V) and membrane LN (MLN; class V) account for approximately 70% and 20% of all LN cases, respectively (6, 14). Early and accurate diagnosis of LN facilitates implementation of the optimum treatment that can prevent flares and preserve renal function (6, 15). The treatment choice in LN mainly depends on the histological class as well as on the activity and chronicity status (6). For example, current recommendations suggest intense immunosuppressive therapy for the treatment of PLN but not class II LN (5, 6, 16, 17). Therefore, early classification of LN has important implications for the therapeutic regimen and prognostic monitoring.

Currently, renal biopsy, which differentiates pathological classes and defines the severity of renal involvement, is the gold standard for the diagnosis of LN (13, 18). However, conversion between the proliferative and membranous forms of LN is frequent (19, 20), and renal biopsy is an invasive approach that may cause complications (21). Thus, this procedure is not suitable for routine monitoring of disease progression. Proteinuria is a major symptom of LN but cannot be used as a reliable LN marker since any renal impairment other than LN can cause this symptom (13, 22). Thus, markers in biofluids accessible with minimal invasiveness are needed to diagnose LN classes.

Human epididymis protein 4 (HE4), also known as whey acidic protein 4-disulfide core domain 2, is a secreted glycoprotein. Serum HE4 level is considered as a vital biomarker for ovarian cancer (23, 24) and an inflammatory biomarker which is elevated in patients with cystic fibrosis (25) and those with renal fibrosis (26). LN is characterized by renal inflammation that damages renal cells and eventually leads to renal fibrosis (27). However, LN classes differ in renal inflammation and fibrosis levels and may thus also differ in serum HE4 level. Furthermore, serum HE4 level in pediatric patients with SLE has not been investigated.

Hence, this study aimed to assess for the correlation of serum HE4 level with adult and pediatric LN classes.

## Materials and methods

### Study design, patients and controls

This study is a retrospective, single-center study. Blood samples were collected from 182 patients with SLE into serum tubes (tubes without anticoagulant) during the first visit at the First Affiliated Hospital of Sun Yat-sen University. The normal control population consisted of 190 healthy adult subjects who received routine physical examination. The diagnosis of SLE was based on the American College of Rheumatology classification criteria (28), and the patient cohort included 100 SLE patients with LN (61 adult-onset LN [aLN]; 39 childhood-onset LN [cLN]) and 82 adult-onset SLE (aSLE) patients without LN. LN was diagnosed based on renal biopsy results. SLE patients complicated with myositis, primary Sjogren’s syndrome, systemic sclerosis or rheumatoid arthritis were excluded. This study was conducted in accordance with the Declaration of Helsinki and approved by the Ethics Committee of the First Affiliated Hospital of Sun Yat-sen University (No. IIT-2021-778).

### Renal pathology

Each biopsy contained > 10 glomeruli and was interpreted by two pathologists (HX and FW) based on 2003 ISN/RPS classification (class I, minimal mesangial LN; class II, mesangial proliferative LN; class III, focal LN; class IV, diffuse segmental or global LN; class V, membranous LN; and class VI, advanced sclerosing LN) (13). For the activity and chronicity assessment, class III and IV LN are sub-classified as LN with purely active (A), purely chronic (C), or mixed (A/C) lesions. Patients with class I or VI LN were absent in this study and thus were not analyzed.

The patients were also categorized as PLN (all the class III, IV, III+V, and IV+V patients) and non-proliferative LN (non-PLN; class II and class V patients), and the PLN patients were sub-classified into pure PLN (class III and class IV patients) and mixed PLN (class III+V and class IV+V patients).

### Data collection and serum HE4 quantitation

Demographic and clinical characteristics and laboratory findings were collected on the day of renal biopsy and comprised information about age, gender, body mass index, and hematological, biochemical, and immunological test results. Serum HE4 level was measured using Architect HE4 kits and an Abbott ARCHITECT i2000SR Immunoassay Analyzer (Abbott Laboratories, Abbott Park, IL, USA) according to the manufacturer’s instructions. Briefly, a two-step immunoassay involving the chemiluminescent microparticle immunoassay technology with flexible assay protocols (Chemiflex) was used.

### Statistical analysis

Statistical analysis was performed using the SPSS software version 26 and GraphPad Prism version 9.1.0. Data were expressed as median values with interquartile ranges (IQRs) for continuous variables and as proportions for categorical variables. The Student’s *t*-test, Mann-Whitney U test, and Chi-squared or Fisher’s exact test were used to analyze normally distributed, non-normally distributed, and categorical data, respectively. Univariate and multivariate logistic regression models were used to assess for the association between serum laboratory findings and aLN. Receiver operating characteristic (ROC) curve was used to evaluate the sensitivity, specificity, and area under the curve (AUC). The cutoff value was determined using the optimal Youden index (sensitivity + specificity –1). Correlation was analyzed using the Spearman rank correlation test. All the *P*-values were two-sided, and a *P*-value < 0.05 was considered to indicate statistical significance.

## Results

### Serum HE4 level was elevated in the patients with aLN

**Figure 1:**
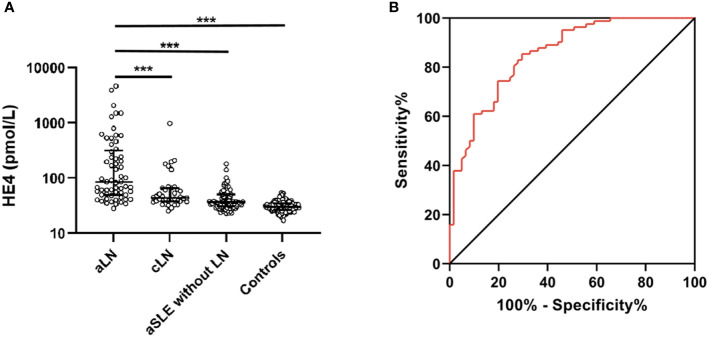
Serum HE4 level in the patients and controls. **(A)** Serum HE4 level is significantly higher in the aLN patients than in other groups. **(B)** ROC analysis of serum HE4 level in distinguishing the aLN cases from the aSLE without LN cases. HE4, human epididymis protein 4; aLN, adult-onset lupus nephritis; ROC, receiver operating characteristic; aSLE without LN, adult-onset systemic lupus erythematosus without lupus nephritis; cLN, childhood-onset lupus nephritis. ***, *P*<0.001.

The characteristics of the patients with LN are shown in **Table 1**. The highest median serum HE4 level was observed in the patients with aLN (median, 85.5 pmol/L; IQR, 49.5–314.7) compared with the levels in the patients with cLN (median, 44 pmol/L; IQR, 38–63.8) and in those with aSLE without LN (median, 37 pmol/L; IQR, 30.5–50.6) as well as in the healthy controls (median, 30 pmol/L; IQR, 26.6–34.7) (**Figure 1A**). ROC analysis revealed that the AUC for HE4 was 0.854 (95% CI 0.793–0.916, *P* < 0.001) to distinguish aLN from aSLE without LN (sensitivity, 70.5%; specificity, 85.4%; cutoff, 57.1 pmol/L) (**Figure 1B**). In addition, the association between the variables and aLN was assessed using univariate and multivariate analyses. Multivariate analysis showed that serum HE4 and IgG levels were significantly associated with aLN in the patients with aSLE (**Table 1**).

**Table 1:** Univariate and multivariate analyses of the variables associated with aLN.

	aLN (N = 61) % (n/N) or median (IQR)	aSLE without LN (N = 82) % (n/N) or median (IQR)	cLN (N = 39) % (n/N) or median (IQR)	Univariable *P* value (aLN *versus* aSLE without LN)	Multivariable^*^ *P* value (aLN *versus* aSLE without LN)
HE4, pmol/L	85.5 (49.5–314.7)	37 (30.5 – 50.6)	44 (38–63.8)	< 0.001	0.031
Demographics					
Age, years	32 (25.5–42)	35 (31.8 – 48.3)	13 (11–13)	0.037	0.090
Gender, female	83.6 (51/61)	89 (73/82)	87.2 (34/39)	0.348	
Body mass index, kg/m^2^	22.3 (19.7–24.5)	20.9 (19.6 – 23.1)	20.6 (17.9–22)	0.538	
Whole blood					
WBC count, ×10^9/L	6.9 (5.4–8.5)	5.4 (4.3–6.7)	6.9 (5.7–9.8)	0.001	0.225
Lymphocyte count, ×10^9/L	1.2 (0.8–1.7)	1.2 (0.9–1.7)	2 (1.5–2.5)	0.934	
Haematocrit, %	0.36 (0.3–0.39)	0.38 (0.35–0.4)	0.4 (0.36–0.42)	0.006	0.862
Platelet count, g/L	220 (180–268)	232 (189–279)	305 (258.5–337)	0.727	
Hemoglobin, g/L	115 (97.5–129)	126 (113.8–135.3)	131 (117.5–136.5)	0.011	0.601
Serum					
Serum C3 level, g/L	0.6 (0.5–0.8)	0.7 (0.5–0.8)	0.8 (0.7–1)	0.231	
Serum C4 level, g/L	0.15 (0.12–0.21)	0.14 (0.1–0.18)	0.13 (0.09–0.18)	0.092	
Serum albumin, g/L	33.8 (27.4–38)	40.7 (38.3–42.4)	42.3 (37.4–44.3)	< 0.001	0.403
Blood urea nitrogen, mmol/L	6.8 (4.8–10.8)	4 (3.3–5.1)	4.5 (3.7–6.53)	0.001	0.416
Serum creatinine, μmol/L	80 (59.5–151.5)	62 (55.3–73)	47.5 (41.3–58)	0.001	0.325
eGFR, mL/min/1.73m^2^	63.9 (33.34–103.09)	93.3 (77–115.6)	137.7 (110.8–179.4)	0.028	0.466
Serum IgG, mg/dl	8.8 (5.9–12.8)	14 (11.8–17.2)	9.64 (6.3–11.7)	< 0.001	0.047
Serum IgA, mg/dl	2 (1.3–2.5)	2.6 (2–3.7)	1.72 (1.1–2.3)	< 0.001	0.843
Serum IgM, mg/dl	0.7 (0.5–1.1)	1 (0.7–1.4)	0.8 (0.5–1.1)	0.003	0.294
Autoantibodies					
ANA, U/mL	93.3 (12.1–300)	244.9 (24–300)	23.2 (11.6–211.8)	0.072	
Anti-dsDNA, IU/mL	37.7 (6.2–104.8)	19.4 (5.5–134.5)	18.6 (5.1–137.3)	0.986	
Anti-Jo-1	0	1.4 (1/72)	0	1	
Anti-Sm	18.6 (11/59)	20.8 (15/72)	20 (7/35)	0.755	
Anti-Ro/SSA	54.2 (32/59)	63.9 (46/72)	37.1 (13/35)	0.264	
Anti-La/SSB	16.9 (10/59)	22.2 (16/72)	11.4 (4/35)	0.453	
Anti-RNP	23.7 (14/59)	44.4 (32/72)	28.6 (10/35)	0.015	0.853
Anti-Scl-70	0	1.4 (1/72)	1 (2.9/35)	1	
Anti-centromere B	1.7 (1/59)	2.8 (2/72)	0	0.683	

^*^ Variables significant on univariate analysis were included in the multivariate logistic regression. aLN, adult-onset lupus nephritis; aSLE without LN, adult-onset systemic lupus erythematosus without lupus nephritis; IQR, interquartile range; HE4, human epididymis protein 4; WBC, white blood cell; C3, complement C3; C4, complement C4; ANA, anti-nuclear antibody.

### Serum HE4 level was elevated in the patients with adult-onset PLN

**Figure 2:**
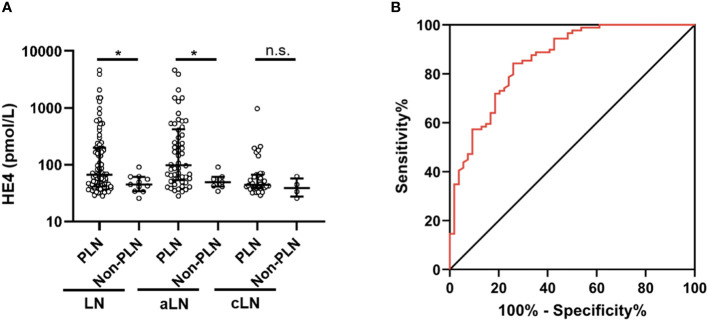
Comparison of serum HE4 levels between the PLN and non-PLN patients **(A)**, and ROC analysis of serum HE4 level in predicting PLN **(B)**. HE4, human epididymis protein 4; PLN, proliferative lupus nephritis; ROC, receiver operating characteristic; aLN, adult-onset lupus nephritis; cLN, childhood-onset lupus nephritis; aSLE without LN, adult-onset systemic lupus erythematosus without lupus nephritis. *, *P*<0.05; n.s., not statistically significant.

A total of 100 patients underwent renal biopsy and were histologically classified (**Table 2**). Serum HE4 level was significantly higher in the PLN patients than in the non-PLN patients, and this difference was found in the aLN (median, 98.3 *versus* 49.3 pmol/L, *P* = 0.021) but not the cLN (median, 44 *versus* 39.1 pmol/L, *P* = 0.333) patients (**Figure 2A**). ROC analysis showed that the optimal cut-off value to distinguish the PLN patients from the non-PLN or aSLE without LN patients was estimated to be 57.1 pmol/L, with a sensitivity and specificity of 74.1% and 84.3%, respectively, and an AUC value of 0.858 (95% CI 0.794–0.921, *P* < 0.001) (**Figure 2B**). Since no significant difference in serum HE4 level was found between the pure PLN (classes III and IV) and mixed PLN (classes III+V and IV+V) cases among all the aLN or cLN cases, or between the class III and class IV LN cases (**Figures S1A–C**), the patients demonstrating features of class III and IV LN concomitantly with features of class V LN were categorized as class III and class IV LN patients, respectively, in the subsequent analyses. There was no significant difference in serum HE4 level between the class III and class IV PLN patients (**Figure S1D**).

**Table 2:** Frequencies of the histological classes in the patients with LN.

	LN (N = 100) % (n/N)	aLN (N = 61) % (n/N)	cLN (N = 39) % (n/N)
PLN (III/IV ± V)	89 (89/100)	88.5 (54/61)	89.7 (35/39)
Pure PLN (III, IV)	67 (67/100)	67.2 (41/61)	66.7 (26/39)
III	17 (17/100)	14.8 (9/61)	20.5 (8/39)
IV	50 (50/100)	52.5 (32/61)	46.2 (18/39)
Mix PLN (III/IV + V)	22 (22/100)	21.3 (13/61)	23.1 (9/39)
III + V	8 (8/100)	8.2 (5/61)	7.7 (3/39)
IV + V	14 (14/100)	13.1 (8/61)	15.4 (6/39)
Non-PLN (II, V)	11 (11/100)	11.5 (7/61)	10.3 (4/39)

LN, lupus nephritis; aLN, adult-onset lupus nephritis; cLN, childhood-onset lupus nephritis; IQR, interquartile range; PLN, proliferative lupus nephritis.

### The class IV aLN patients with A/C lesions had especially high serum HE4 levels

**Figure 3:**
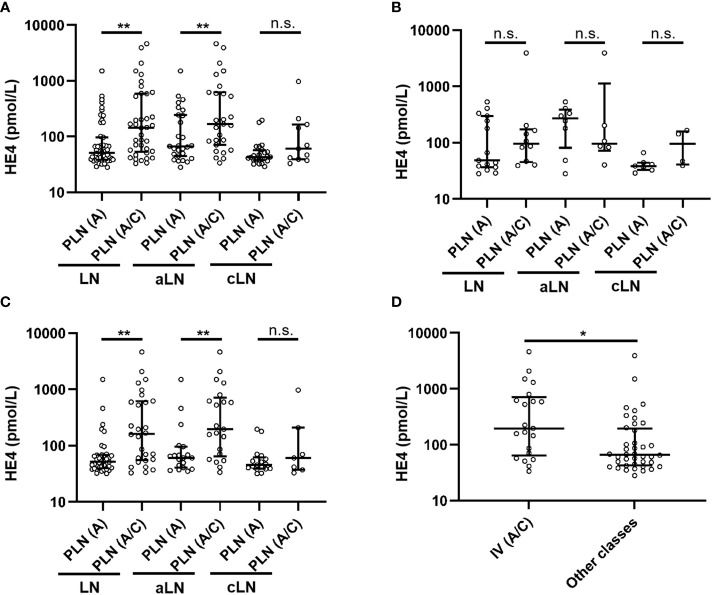
Comparison of serum HE4 levels between A and A/C lesions in the patients with class III/IV LN **(A)**, class III LN alone **(B)**, and class IV LN alone **(C)**, as well as between the aLN patients with class IV (A/C) and those with other classes **(D)**. HE4, human epididymis protein 4; A, purely active lesions; A/C, active and chronic lesions; LN, lupus nephritis; PLN, proliferative lupus nephritis; aLN, adult-onset lupus nephritis; cLN, childhood-onset lupus nephritis. *, *P*<0.05; **, *P*<0.01; n.s., not statistically significant.

As shown in **Table 3**, of the 89 patients with class III/IV LN, 51 had A lesions, and 38 had a combination of A and C lesions. Overall, the patients with class III/IV (A) LN had significantly lower serum HE4 levels than those with class III/IV (A/C) LN (class III/IV [A] *versus* class III/IV [A/C]: median, 51.1 *versus* 143.5 pmol/L, *P* = 0.001). This difference was found only in the aLN cases but not in the cLN cases (**Figure 3A**). When stratified by LN class, no significant difference in serum HE4 level was found between the class III (A) and class III (A/C) aLN patients (**Figure 3B**), whereas serum HE4 level was significantly lower in the class IV (A) aLN patients than in those with class IV (A/C) aLN (class IV [A] *versus* class IV [A/C]: median, 60.8 *versus* 195.5 pmol/L, *P* = 0.006) (**Figure 3C**). The patients with class IV (A/C) aLN had significantly higher serum HE4 levels than those with any other aLN class (median, 195.5 *versus* 65.7 pmol/L; *P* = 0.009) (**Figure 3D**).

**Table 3:** Serum HE4 level in the PLN patients with A or A/C lesions.

	PLN (N = 89) % (n/N) or median (IQR)			aPLN (N = 54) % (n/N) or median (IQR)			cPLN (N = 35) % (n/N) or median (IQR)		
	n	HE4	*P* value	n	HE4	*P* value	n	HE4	*P* value
III/IV (A)	51	51.1 (38.5–96)	0.001	27	66.7 (44.7–243.9)	0.024	24	42.6 (37.9–56.7)	0.133
III/IV (A/C)	38	143.5 (53.4–586.2)		27	166.5 (70.7–622.5)		11	60.3 (39.2–164.1)	
III (A)	15	48.4 (36.6–297.6)	0.531	8	270.8 (81.1–387)	0.491	7	38.5 (32.6–44)	0.174
III (A/C)	10	96.4 (45–173.3)		6	96.4 (71.9–1128)		4	94.7 (41.1–158.8)	
IV (A)	36	52.1 (39.6–68.1)	0.002	19	60.8 (40.3–96)	0.006	17	45.2 (38.8–62)	0.494
IV (A/C)	28	162 (55–615)		21	195.5 (63.9–708.9)		7	60.3 (37.1–208)	

HE4, human epididymis protein 4; PLN, proliferative lupus nephritis; A, purely active lesions; A/C, active and chronic lesions; aPLN, adult-onset proliferative lupus nephritis; cPLN, childhood-onset proliferative lupus nephritis; IQR, interquartile range.

### Correlations between serum HE4 level and various parameters

The patients with class IV aLN were assessed for any correlation between their serum HE4 levels and various variables. In these patients, significant positive correlations were found between serum HE4 level and blood urea nitrogen, serum creatinine, and 24-hour and random proteinuria levels, and significant negative correlations were found with serum albumin, hemoglobin, and complement C3 and C4 levels, hematocrit value, and absolute lymphocyte count (**Table 4**, **Figures S2A–J**). No significant correlation was seen between serum HE4 level and the other parameters analyzed (**Table S1**).

**Table 4:** Bivariate correlations of serum HE4 level with the variables in the patients with class IV aLN.

	r	95% CI	*P* value
Blood urea nitrogen	0.765	0.541 – 0.888	< 0.001
Serum creatinine	0.754	0.522 – 0.882	< 0.001
24-hour proteinuria	0.513	0.126 – 0.765	0.010
Random proteinuria	0.421	0.045 – 0.692	0.026
Serum albumin	-0.667	-0.836 – -0.382	< 0.001
Hemoglobin	-0.660	-0.832 – -0.370	< 0.001
Haematocrit	-0.644	-0.824 – -0.346	< 0.001
Serum C3 level	-0.494	-0.737 – -0.136	0.008
Lymphocyte count	-0.407	-0.684 – -0.029	0.032
Serum C4 level	-0.398	-0.678 – -0.018	0.036

HE4, human epididymis protein 4; aLN, adult-onset lupus nephritis; CI, confidence interval; C3, complement C3; C4, complement C4.

## Discussion

Previous studies have revealed that serum HE4 level is a risk factor for developing in LN among adult patients with SLE (29, 30). Given that LN includes various classes and serum HE4 level in pediatric patients with SLE has not been investigated, the present study focused on serum HE4 level in patients with different aLN or cLN classes. Here, we revealed that serum HE4 and IgG levels were independently associated with aLN. The reason why patients with aLN included in this study had lower levels of IgG compared to SLE patients without LN may be that they were more likely to have been treated with immunosuppressants that led to a reduced synthesis of IgG. Additionally, we demonstrated that serum HE4 level was increased in the PLN subgroup of aLN, especially in class IV (A/C) aLN, and observed a significant association between serum HE4 levels and renal functions measured by blood urea nitrogen (BUN) and serum creatinine levels. However, the association between serum HE4 and SLE disease activity index (SLEDAI) was not available to be analyzed due to the lack of SLEDAI data.

The pathogenic mechanism of LN is not completely understood. To date, LN has been thought to be initiated by the immune complexes and complement components in the glomeruli, and its pathogenesis involves continuing inflammation, hypoxia, metabolic abnormalities, aberrant tissue repair, and tissue fibrosis (31–33). PLN is a frequent and severe type of LN and entails a more aggressive course and deterioration of renal function and higher risk of progression to ESRD than non-PLN (6, 14, 19, 34). Patients with PLN have poor early response to treatment (within 6 months) and poor outcomes (35, 36). Effective clinical management of PLN is vital for maximal renal survival in patients with this disease and highly dependent on accurate and timely diagnosis and therapy. Thus, early laboratory parameters of (non-)response to induction treatment and of high risk of poor renal outcome may prove beneficial in determining the optimum treatment choice. Conventional parameters, such as serum creatinine level and proteinuria, are neither sensitive nor specific for differentiating LN from other glomerulopathies or distinguishing active inflammation from chronic scarring in the kidneys and do not accurately reflect histopathological changes (37). In this study, we found that serum HE4 level is increased in adult patients with PLN, indicating that serum HE4 level can be a promising non-invasive diagnostic biomarker of PLN. Though serum HE4 level was not significantly higher in childhood patients with PLN, this may be explained by the relatively small sample size of childhood patients, thus a larger sample size of childhood proliferative LN patients are needed to analyze the difference in serum HE4 levels when stratified by LN class. Overall, this finding provides a biofluid-based diagnostic method complementary to renal biopsy in PLN. Besides PLN, the relationship between kidney involvement and serum HE4 level were also found in primary Sjogren’s syndrome (38) and systemic sclerosis (39).

Class III lesions were defined as proliferative glomerulonephritis affecting fewer than 50% of the glomeruli, whereas class IV was defined as proliferative glomerulonephritis affecting more than 50% of the glomeruli. In this study, we further subdivided PLN into A and A/C subgroups based on renal histopathological features. We found that patients with class IV (A/C) aLN have significantly higher serum HE4 levels than those with class IV (A) aLN, indicating that HE4 might be involved in C lesions. However, there was no significant difference in serum HE4 level between the class III (A/C) and class III (A) aLN patients. These results may be caused by the difference in the severity of the lesions between class IV and class III. Furthermore, since PLN patients with A/C lesions have significantly higher severity scores of interstitital fibrosis than those with A lesions (40), HE4 might be involved in interstitial fibrosis. A positive association between serum HE4 level and renal fibrosis has been reported (41, 42). Myofibroblasts are important mediators of renal fibrosis. LeBleu et al. have revealed that *HE4* is an upregulated gene in myofibroblasts, and it can bind to and inhibit multiple proteases, including serine proteases and matrix metalloproteinases, thereby suppressing the proteolytic degradation of type I collagen (26). Neutralization of HE4 accelerates collagen I degradation and alleviates renal fibrosis in mouse models of renal diseases (26). Our study provides an additional insight for a better understanding of the pathogenesis of C lesions in PLN. Accordingly, HE4 might be a potential therapeutic target for the treatment of PLN. Nevertheless, the role of HE4 in PLN should be investigated further in the future.

The composition of urine, containing waste products from blood that are filtered and excreted by kidneys, can reflect the state of renal function. However, we did not measure urine HE4 levels of the patients. In addition, sera from patients with other renal diseases could be collected to detect serum HE4 level. Thus, a prospective cohort study should be designed to concurrently collect serum and urine samples from patients and analyze whether urine HE4 level can predict the diagnosis of PLN.

## Conclusion

Serum HE4 level is elevated in adult patients with PLN, and HE4 may play a role in the pathogenesis of chronic lesions in patients with class IV aLN.

## Data availability statement

The raw data supporting the conclusions of this article will be made available by the authors, without undue reservation.

## Ethics statement

The studies involving human participants were reviewed and approved by the Ethics Committee of the First Affiliated Hospital of Sun Yat-sen University (No. IIT-2021-778). Written informed consent from the participants’ legal guardian/next of kin was not required to participate in this study in accordance with the national legislation and the institutional requirements.

## Author contributions

Conceptualization: LBL, LSL, and ML. Study design: LBL, FW, and HZhang. Sample collection: LBL, HYX, YL, RL, QS, HZhu, HXX, FW, and HZhang. Data acquisition: LBL, HYX, YL, RL, QS, and HZhu. Data analysis and interpretation: LBL, HYX, HXX, LSL, ML, FW, and HZhang. Statistical analyses: LBL, and RL; funding acquisition: LBL. Original draft preparation: LBL, and HYX. Manuscript review and editing: FW, and HZhang. Supervision: HXX, ML, FW, and HZhang. All authors contributed to read and approved the final version of the manuscript. Authors take full responsibility for all aspects of the presented work.
