# Supplementary material for: Elevated serum levels of human epididymis protein 4 in adult patients with proliferative lupus nephritis
Source: Front Immunol. 2023 May 23;14:1179986. doi: 10.3389/fimmu.2023.1179986 (PMC10243370; doi:10.3389/fimmu.2023.1179986)

**Table S1** Bivariate correlations of serum HE4 level with other parameters in the patients with class IV aLN

|  | r | 95% CI | *P* value |
| --- | --- | --- | --- |
| Urine cast | 0.258 | -0.1383 to 0.5836 | 0.184 |
| Serum IgM | 0.129 | -0.2675 to 0.4877 | 0.514 |
| Anti-dsDNA | 0.111 | -0.2843 to 0.4737 | 0.575 |
| Age | 0.103 | -0.2915 to 0.4676 | 0.602 |
| WBC count | 0.085 | -0.3079 to 0.4534 | 0.667 |
| Serum IgA | -0.340 | -0.6396 to 0.04956 | 0.077 |
| Gender | -0.240 | -0.5705 to 0.1575 | 0.219 |
| Platelet count | -0.231 | -0.5641 to 0.1668 | 0.237 |
| Urine WBC | -0.193 | -0.5363 to 0.2052 | 0.325 |
| Anti-RNP | -0.171 | -0.5321 to 0.2435 | 0.405 |
| Anti-Sm | -0.171 | -0.5321 to 0.2435 | 0.405 |
| Anti-Ro/SSA | -0.134 | -0.5045 to 0.2787 | 0.515 |
| Serum IgG | -0.134 | -0.4914 to 0.2629 | 0.498 |
| Body mass index | -0.102 | -0.5314 to 0.3686 | 0.668 |
| ANA | -0.089 | -0.4562 to 0.3047 | 0.654 |
| Urine RBC | -0.025 | -0.4044 to 0.3612 | 0.898 |
| Anti-La/SSB | -0.019 | -0.4137 to 0.3812 | 0.926 |

HE4, human epididymis protein 4; aLN, adult-onset lupus nephritis; CI, confidence interval; C4, complement C4; C3, complement C3; WBC, white blood cell; ANA, anti-nuclear antibody; RBC, red blood cell.

**Fig. S1** Comparison of serum HE4 levels between pure PLN and mixed PLN in the patients with class III/IV LN (**A**), class III LN alone (**B**), and class IV LN alone (**C**), as well as between classes III and IV LN (**D**). HE4, human epididymis protein 4; PLN, proliferative lupus nephritis; LN, lupus nephritis; aLN, adult-onset lupus nephritis; cLN, childhood-onset lupus nephritis.


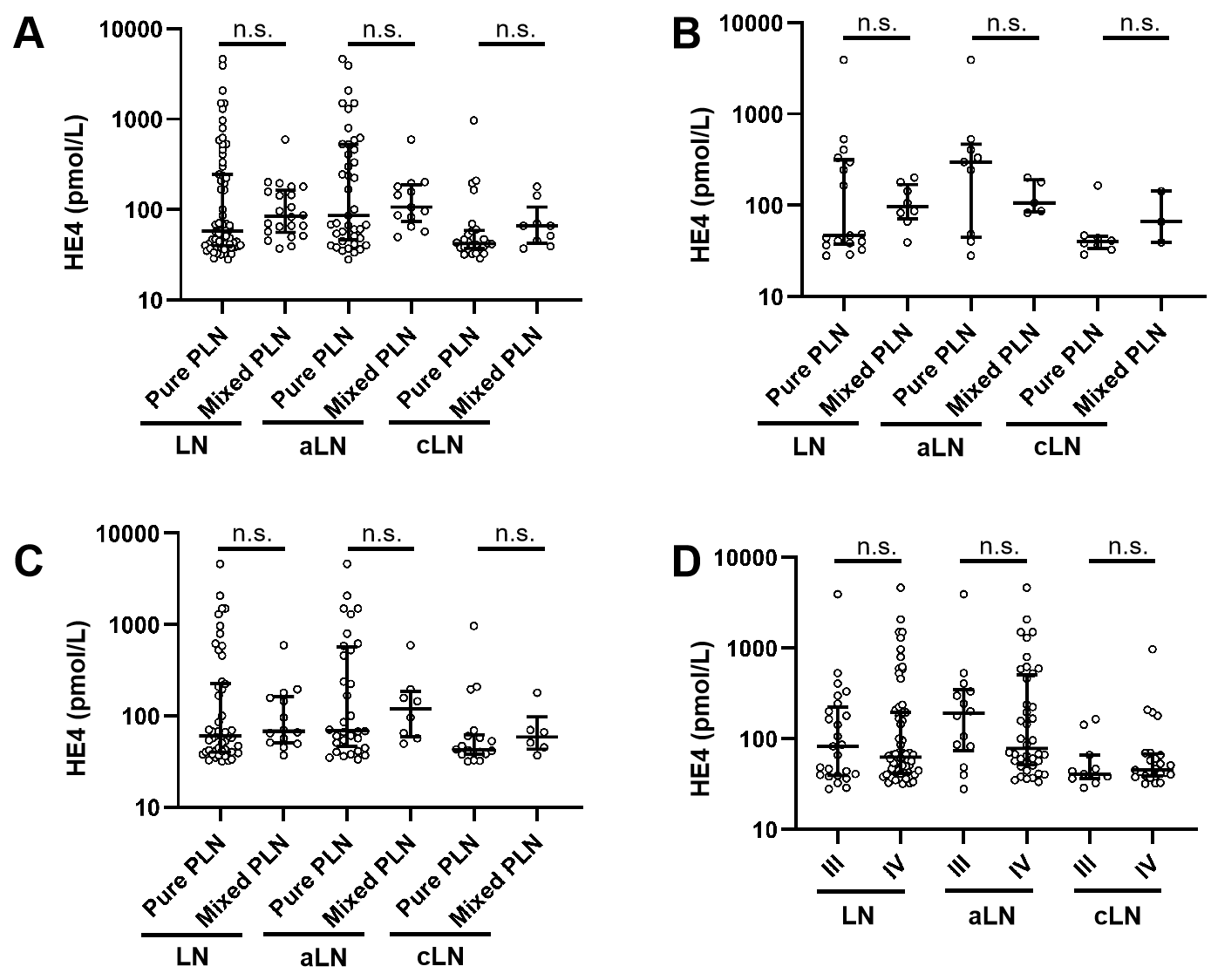


**Fig. S2** Bivariate correlations of serum HE4 level with the variables in the patients with class IV aLN. HE4, human epididymis protein 4; aLN, adult-onset lupus nephritis; C3, complement C3; C4, complement C4.


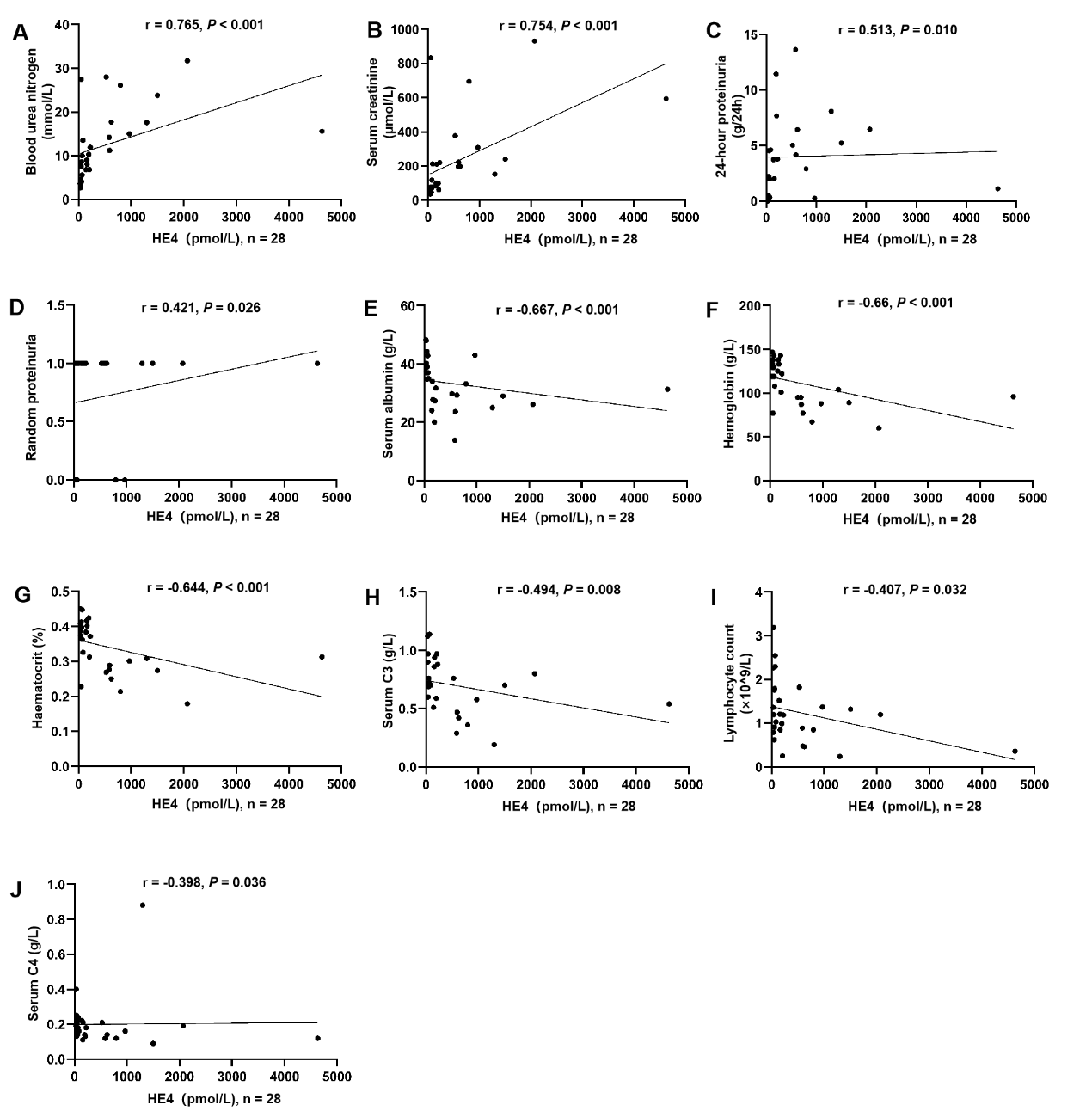

Supplement: Supplementary file 1 [file DataSheet_1.docx]
